# Improving zebrafish embryo xenotransplantation conditions by increasing incubation temperature and establishing a proliferation index with ZFtool

**DOI:** 10.1186/s12885-017-3919-8

**Published:** 2018-01-02

**Authors:** Pablo Cabezas-Sainz, Jorge Guerra-Varela, María J. Carreira, Javier Mariscal, María Roel, Juan A. Rubiolo, Andrés A. Sciara, Miguel Abal, Luis M. Botana, Rafael López, Laura Sánchez

**Affiliations:** 1 Departament of Zoology, Genetics and Physical Anthropology, Universidade de Santiago de Compostela, Lugo, Spain; 2 Investigation Center of Information Technologies (CiTIUS), Universidade de Santiago de Compostela, Santiago de Compostela, Spain; 3 Translational Laboratory, Medical Oncology Department, Complexo Hospitalario Universitario de Santiago de Compostela/SERGAS, Santiago de Compostela, Spain; 4 Department of Pharmacology, Veterinary Faculty, Universidade de Santiago de Compostela, Lugo, Spain; 5 Molecular and Cellular Biology Institute of Rosario (IBR-COCINET) – Biochemistry and Pharmaceutics Science Faculty, National Rosario University, Rosario, Santa Fe, Argentina

**Keywords:** Zebrafish, Xenograft, Cancer, 5-fu, Proliferation, Temperature, ZFtool

## Abstract

**Background:**

Zebrafish (*Danio rerio*) is a model organism that has emerged as a tool for cancer research, cancer being the second most common cause of death after cardiovascular disease for humans in the developed world. Zebrafish is a useful model for xenotransplantation of human cancer cells and toxicity studies of different chemotherapeutic compounds in vivo. Compared to the murine model, the zebrafish model is faster, can be screened using high-throughput methods and has a lower maintenance cost, making it possible and affordable to create personalized therapies. While several methods for cell proliferation determination based on image acquisition and quantification have been developed, some drawbacks still remain. In the xenotransplantation technique, quantification of cellular proliferation in vivo is critical to standardize the process for future preclinical applications of the model.

**Methods:**

This study improved the conditions of the xenotransplantation technique – quantification of cellular proliferation in vivo was performed through image processing with our ZFtool software and optimization of temperature in order to standardize the process for a future preclinical applications. ZFtool was developed to establish a base threshold that eliminates embryo auto-fluorescence and measures the area of marked cells (GFP) and the intensity of those cells to define a ‘proliferation index’.

**Results:**

The analysis of tumor cell proliferation at different temperatures (34 °C and 36 °C) in comparison to in vitro cell proliferation provides of a better proliferation rate, achieved as expected at 36°, a maintenance temperature not demonstrated up to now. The mortality of the embryos remained between 5% and 15%. 5- Fluorouracil was tested for 2 days, dissolved in the incubation medium, in order to quantify the reduction of the tumor mass injected. In almost all of the embryos incubated at 36 °C and incubated with 5-Fluorouracil, there was a significant tumor cell reduction compared with the control group. This was not the case at 34 °C.

**Conclusions:**

Our results demonstrate that the proliferation of the injected cells is better at 36 °C and that this temperature is the most suitable for testing chemotherapeutic drugs like the 5-Fluorouracil.

**Electronic supplementary material:**

The online version of this article (10.1186/s12885-017-3919-8) contains supplementary material, which is available to authorized users.

## Background

Model organisms are very important for understanding human diseases [1]. Of the current available vertebrate animal models, genetic and experimental zebrafish and mouse models have contributed significantly to advancing our insights into cancer biology and therapy [2], largely due to the high genomic similarities they share with humans [3].

Zebrafish, described for the first time by George Streisinger, emerged as a model organism for developmental genetics in the 1960s [4]. Since then, the zebrafish model has been extensively used in biomedical studies for several reasons, which include the large numbers of descendants, small physical size, reduced cost of maintenance, availability of genetically modified lines, embryo transparency and, most importantly, the small amount of drug required for testing new compounds in drug screening assays [5].

The capacity of tumors to efficiently engraft in animals has been known since the first experiments were conducted in mice in the 19XXs [6]. Tumor transplantation in animal models is very informative; not only can it provide data on tumor growth and the metastatic potential of tumor cells, but it also offers the possibility to test drugs in an in vivo animal setting, which could be putatively applied to the clinical setting to determine the best treatment for patients [7]. While these types of studies have been carried out for nearly half a century with mouse models, it was not until 2006 that Haldi et al. [8] performed the first xenograft of leukemia cells in zebrafish, highlighting the potential use of this alternate vertebrate animal for tumor-based studies. Since then, several studies have demonstrated that zebrafish are an excellent model for the transplantation of tumor cell lines or primary patient derived cells [9–12]. Specifically, with zebrafish there are three opportunities available for tumor cell transplantation: 1) the embryo stage, when the innate immune system is present, but the acquired immune system has yet to be fully developed, 2) transplantation in adult fish lines, such as Casper, with rejection inhibiting treatments [13] or 3) the recently developed immunocompromised adult rag2^E450fs^ mutant zebrafish [12, 14].

The most commonly used methodology for cancer xenograft assays in zebrafish consists in real time tracking of tumor cells (labeled with different fluorescent dyes [15] or that constitutively express a fluorescent protein in the cytoplasm) introduced via microinjection into particular zones of 48 h-old zebrafish embryos. Tumor cell proliferation and their invasive capacity can then be analyzed over the course of several days [16].

When compared to the murine xenotransplantation model, the zebrafish model offers several advantages, including the high number of embryos that can be injected, allowing for statistical analysis of the aforementioned parameters in a few days; the possibility to test several concentrations/combinations of drugs in 96 well plate formats [9, 17]; and the lack of an acquired immune system in embryos [18], which facilitates tumor cell engraftment.

While a promising model, several drawbacks need to be considered when using zebrafish for xenotransplantation assays. One of the most important [19] limitations is the temperature (28 °C) at which these fish are routinely maintained, which differs by 9 degrees from that of the human body (37 °C), the latter being the ideal temperature for tumor cell proliferation. To tackle this problem, several groups have described incubation temperatures for xenografts in zebrafish ranging from 31 °C to 34 °C [see Additional file 1: Figure S6], as a compromise solution between the optimal temperature for human cell proliferation and zebrafish survival.

The analysis of cellular proliferation inside the embryo is another challenge considering the high number of fish that need to be imaged in high resolution, and the short period of time available to test different compounds and examine the effect on the injected cells [20, 21]. Different image analyses can be performed using commercial and free software to estimate the number of cells at the beginning and at the end of the experiment [22, 23], but these techniques are not accurate enough to reliably measure the proliferation of the cells as they are dependent on user intervention in terms of manually adjusting parameters for each image. In this work, we introduce the software ZFtool, which addresses the current problems faced in zebrafish imaging as the features used to extract the proliferation index (area and mean intensity of GFP points) with ZFtool are automatically computed and adapted to the autofluorescent characteristics of each fish. In this way, the measurements are repeatable, reproducible and reliable without user intervention. Performing the necessary computations on a fish-by-fish and stage-by-stage level, and manually adjusting all the parameters results in data that are difficult to compare leading to unreliable results. To provide a solution to this inherent problem, we developed, implemented and validated the automatic ZFtool methodology as described below. At this moment, the software is a Matlab toolbox and the software interface is currently under development.

To significantly improve the technique of assaying different chemotherapeutic agents in an in vivo system, at a temperature almost equal to that of the human body, and in a fast and efficient way, in this study we present a zebrafish yolk xenotransplantation assay together with an image analysis software that provides an answer to the main problems currently faced in the zebrafish xenotransplantation community. Tumor cell injection and rearing conditions were established so that experiments were performed at 36 °C, a temperature that to our knowledge has not been reported before for this type of assay. The conditions utilized showed a good overall survival rate of the embryos, facilitated tumor growth, and together with the automated measurements obtained with the new ad-hoc imaging analysis software ZFtool, we were able to accurately monitor tumor growth with high reproducibility in order to generate reliable results.

## Methods

### Zebrafish handling

The care, use and treatment of zebrafish were performed in agreement with the Animal Care and Use Committee of the University of Santiago de Compostela and the standard protocols of Spain (Directive 2012-63-UE). The protocol was approved by the Animal Care and Use Committee of the University of Santiago de Compostela. One-year-old adult zebrafish (*Danio rerio*, wild-type) were maintained at 28.5 °C in 30 L aquaria at a rate of 1 fish per liter of water, with a light-dark cycle of 14:10. Zebrafish embryos were obtained from mating adults according to previously described procedures [24]. When needed, embryos were euthanized by tricaine overdose.

### Reagents and cell culture

The human colorectal cancer cell line HCT116 was obtained from American Type Culture Collection (ATCC, Catalog No. CCL-247) and cultured using McCoy’s 5A Medium containing 10% FBS (GIBCO, Invitrogen) and 1% Pen/Strep (GIBCO, Invitrogen) at 37 °C with 5% CO_2_ in a humidified atmosphere. The HCT116 cell line was transfected to express GFP constitutively. The HCT116 line was tested monthly for contamination.

### Fluorescent GFP cell labeling

HCT116 cells were transduced using a lentiviral-driven GFP construct (Sigma, Mission TurboGFP, SHC003 V). Cells were placed 72 h post infection under selective pressure using 10 μg/ml puromycin. The rate of GFP positive cells was tested using flow cytometry (BD FACS Aria I, software FACSDiva 6.0.3).

### Cell proliferation assays

Cell proliferation was determined using xCELLigence Real-Time Cell Analyzer; Acea Biosciences (Roche) following the manufacturer instructions. In brief, cells were seeded on E-plates containing electric nodes in their surface that allow the measurement of changes in impedance attributed to cell proliferation. Measurements were performed in quadruplicate, normalizing the initial cell index once the cells were completely adhered.

### Cell injection

Two days post fertilization (dpf), zebrafish embryos were dechorionated (if needed) and anesthetized with 0.003% tricaine (Sigma). Cells were suspended at 10,000-20,000 cells/μl in complete McCoy and maintained at room temperature for no longer than 2 h before they were injected. The cell suspension was loaded into borosilicate glass capillary needles (1 mm O.D. × 0.78 mm I.D.; Harvard Apparatus), and injections were performed using IM-31 Electric Microinjector (Narishige) with an output pressure of 34 kPa and 30 ms injection time. The injections were performed manually right into the yolk of the embryo. Incorrectly injected embryos without cells inside of the yolk, or showing them in the circulation after xenotransplantation were discarded.

### Incubation, imaging and cell quantification

After injection, 2dpf embryos were incubated at two different conditions (34 °C or 36 °C) in 24-well plates with salt dechlorinate tap water (SDTW, chlorine free water obtained with a reverse osmosis filter system) for 72 h to check the proliferation of the cell line by ZFtool. Each plate contained at least 2 negative controls (injected with complete McCoy medium) and 2 blanks (not injected). Apart from those plates, another plate with 12 negative controls and 12 blanks were included in some experiments to test the viability of the embryos. No development abnormalities were observed during incubation at this temperature.

In order to reach a 36 °C incubation temperature without a large amount of embryo mortality, plates were covered with a transparent sealing tape (PCR Plastics) to prevent evaporation and reduction of dissolved oxygen. After that, plates were placed inside an incubator with minimal contact between the plate and the incubator structure to prevent water overheating.

Each embryo was photographed with AZ-100 Nikon fluorescence stereomicroscope at 0 hpi and 72 hpi to be analyzed by ZFtool software. The objective of this software is to automatize and improve the task of measuring the number and mean value of GFP pixels in order to compare them for these two conditions and compute the proliferation index. Finally, this analysis yields the number of GFP pixels in the image (nGFP), which represents the area of the cells inside the yolk sac at two different times and the GFP intensity Medium Value (GMV), which represents the medium intensity of the fluorescence inside the yolk. By multiplying the nGFP number by the GMV of each image, we determined the proliferation ratio between 0 hpi and 72 hpi to estimate the cell growth. The result obtained at 72 hpi was divided by that obtained at 0 hpi, yielding a proliferation index value (PI):

$$ \frac{nGFP_{72 hpi}\bullet {GMV}_{72 hpi}}{nGFP_{0 hpi}\bullet {GMV}_{0 hpi}} $$

A PI value =1 means that cells remain stable during incubation, a PI higher than 1 indicates tumor cell proliferation and a PI lower than 1 indicates tumor cell death.

Zebrafish embryos have variable autofluorescence, especially in the yolk area. To accurately quantify the injected cells fluorescence a pre-processing is needed to only count the GFP pixels belonging to injected cells filtering autofluorescence. To achieve this, the software counts the number of GFP pixels with different intensity thresholds, from 0 (no threshold) to 50 (Fig. 2) and the ZFtool algorithm provides a homogeneous measurement of the GFP area for all fish analyzed comparing nGFP for each threshold analyzed with nGFP for threshold = 0, where fish auto fluorescence is mostly present. When the relation between measured nGFP compared to nGFP at threshold = 0 surpass a fixed value, we consider the GFP area to be stable and the threshold is fixed at this point. In case there is no autofluorescence in the embryo, the threshold is established based on a tolerance parameter and a correction is included to assure the accuracy of the measurement in this cases. The ZFtool algorithm automatic thresholding for each analyzed embryo is one of the main automation components of the software, making it efficient in producing reliable fish to fish measurements.

### Cell counting software

The ZFTool extension for cell counting was developed. A drop of cells was placed on a microscope slide and photographed to obtain a fluorescence image. The algorithm detects circular objects of the fluorescence input image with a fixed diameter. The output yields a fluorescence image with nearly every cell or group of cells delimited by a contour and an estimation of the number of cells inside the input image. This algorithm is based on the circular Hough transform and has several parameters fixing the strength of the edge, and a minimum and maximum radius of the circles to detect. As we know the approximate size of the cells, we can fix these parameters in order to obtain an estimation of the number of cells. The method will be more accurate as the cells are more isolated, but as the number of cells injected increases over 400, we do not need the exact number of cells, but only an estimation, so this method still fits our purposes.

### Anticancer drugs toxicity and treatment

In order to test the toxicity of an anticancer drug (5-Fluorouracil), experiments were performed according to the OECD (Organisation for Economic Co-operation and Development) guideline for the testing of chemicals [25]. This procedure consists of exposing 0 h post fecundation (hpf) eggs to dissolved chemicals in 24-well plates, for a period of 96 h. Various indicators (such as coagulation of embryos, lack of somite formation, non-detachment of the tail or lack of heartbeat) were checked every 24 h during the experiment, to test the mortality of the embryos and calculate the LC50 (lethal concentration 50%) at the end of the test. The drug was tested to determine a concentration range that included 0–100% mortality. Experiments were considered valid when egg fertilization was ≥ 70%. At the beginning, the oxygen concentration should have ≥ 80% saturation, and the water temperature should be 26 ± 1 °C. During the test, the negative control embryos mortality could not be ≥ 10% at any time of the experiment. Exposure to the positive control resulted in a minimum mortality of 30% at the end, and the hatching rate of the negative control embryos was higher than 80 % at 96 h. The concentrations tested were 250 μM, 500 μM, 1000 μM, 1500 μM, 2000 μM, with 1% DMSO. Another analog experiment was conducted changing the treatment starting point from 0 hpf to 48hpf in order to evaluate how the toxicity changed with a dechorionated embryo at 36 °C.

### Statistical analysis

Homoscedasticity and statistical analyses were performed using the SPSS software (IBM). An excel outlier analysis was performed using interquartile range (IQR), while the outliers were discarded. One factor ANOVA for non-parametrical data was applied to non-homoscedastic data with confidence intervals of 95% or 99%, and a Student’s t-test was applied to homoscedastic data with confidence intervals of 95% or 99%. Number of embryos analyzed is represented by n_rep_ and n_total_, being n_rep_ the number of embryos in each replica, and n_total_ the total number of embryos statistically analyzed for the experiment.

## Results

### Fish viability at 34 °C and 36 °C

Data from all experiments were analysed to determine fish viability between 34 °C and 36 °C for 72 h (experimental time range). Both the control (injected with medium) and blank (not injected) groups had a survival rate higher than 95%. Although the data showed that a difference existed between the survival rate at 34 °C (95.37%) and 36 °C (87.5%), statistical analysis found no significant differences (Table 1). At 36,5 °C or above the survival of the embryos is seriously affected and severe deformations were observed (data not shown).

**Table 1:** Total survival percentage of each set of experiments for the zebrafish embryos at three different conditions tested

Experiments	72 h Injected alive	Initial embryos injected
HCT116-GFP 34 °C		
1	12	12
2	47	48
3	44	48
TOTALS	103	108
Survival (% ± SD)	95.370 ± 0.043	
HCT116-GFP 36 °C		
1	42	48
2	18	24
3	24	24
TOTALS	84	96
Survival (% ± SD)	87.500 ± 0.125	
HCT116-GFP 36 °C 5-FU		
1	45	48
2	20	24
3	22	24
TOTALS	87	96
Survival (% ± SD)	90.625 ± 0.055	

Despite the differences observed between the two temperatures, experiments at 36 °C show adequate fish viability in terms of cell proliferation, metabolism and behaviour of the injected cells if we are looking to simulate human body conditions.

### In vitro analysis of HCT116 cell line proliferation

**Fig. 1:**
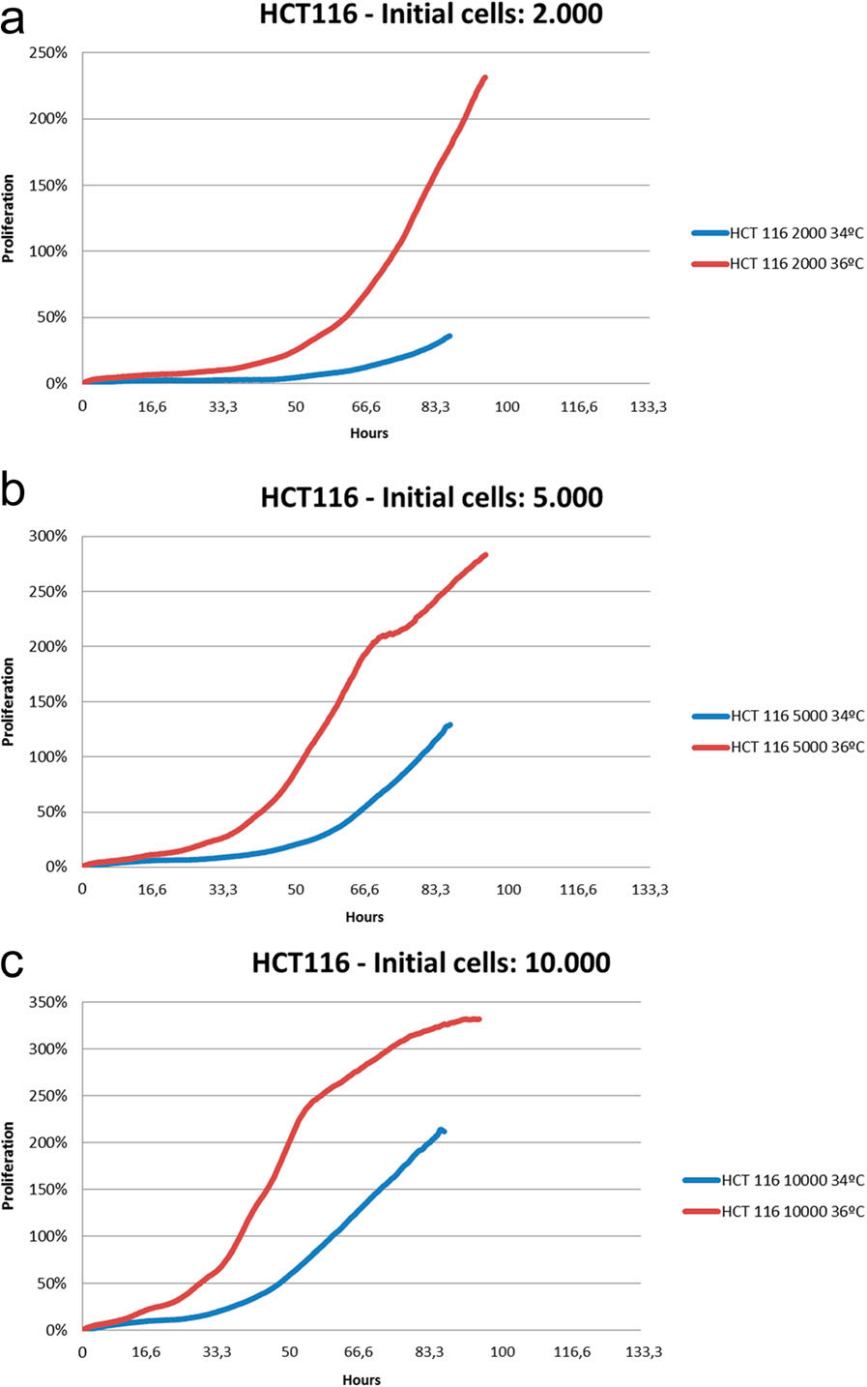
Proliferation of HCT116 cells in vitro. XCELLigence technology was used to quantify the proliferation of the HCT116 cell line in vitro at 34 °C and 36 °C with different initial number of cells (**a**: 2.000, **b**: 5.000 and **c**: 10.000). A better proliferation rate can be observed at 36 °C. The results shown are the media of 4 independent experiments

The Xcelligence technology was used to test in vitro cell proliferation at 34 °C and 36 °C, starting with different initial cell concentrations per well (2.000 cells, 5.000 cells and 10.000 cells). As expected, a better proliferation rate was observed at 36 °C, confirming the data obtained in vivo (Fig. 1).

### Image analysis: ZFtool software

ZFTool software has been designed to provide specific, intuitive and automated tools for zebrafish xenotransplantation and drug testing assays. This software has two main functionalities: cell counting prior to injection and cell proliferation measurement inside the yolk of the embryo. This can be achieved automatically, without programming knowledge in a very intuitive way. Afterwards, other packages could be implemented to enhance the analysis of the proliferating cells, for example a 3D analysis model. ZFTools software is currently being further developed and tested and for that reason is not available for use outside our group. After being thoroughly tested it will be made available for the scientific community.

**Fig. 2:**
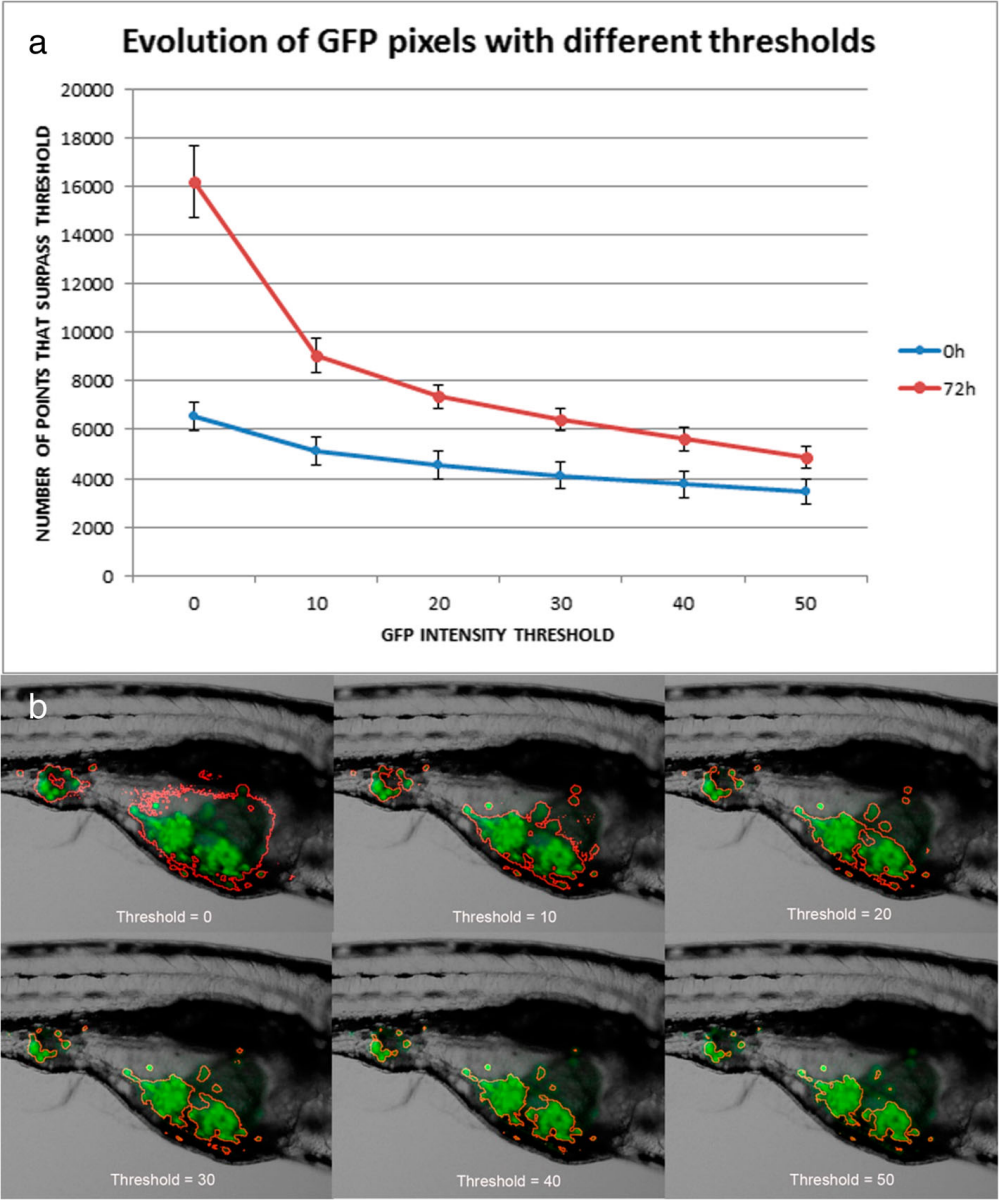
Evolution of the number of GFP pixels based on GFP intensity thresholds for zebrafish embryos and regions of interest of fluorescent zebrafish applied with different thresholds. **a** Graphical representation of average GFP intensity thresholds on the x-axis and mean number of pixels greater than the threshold on the y-axis for the zebrafish embryos tested (*n* = 6). A progressive decay of the area, more evident at 72 hpi (dotted lines), is shown. It can also be observed that as the threshold increases, the area decreases slightly. At a low threshold, auto-fluorescence can represent an important component of GFP intensity. However, as soon as this threshold is raised, auto-fluorescence drastically disappears. Blue line represents 0 hpi embryos, and red line represent 72 hpi embryos. **b** Example of segmentation in evolution with red outlines over the images with thresholds from 0 to 50. The region inside the red outline is reduced as the threshold increases. This way the brightest pixels with higher fluorescence are selected, eliminating the majority of auto-fluorescence from the zebrafish embryo

Image analysis with ZFTool was performed with the parameters established in the code, appropriate to different sets of images taken under different conditions. This tool automatically eliminates fish autofluorescence, as these pixels interfere with the measurement of GFP area [see Additional file 2: Figure S1]. Usually, the darkest GFP pixels correspond to fish autofluorescence, and these pixels must not be included when measuring GFP area and mean intensity. ZFtool automatically establishes a GFP threshold for each fish, taking into account the decay of the graph representing the GFP area at different thresholds. When the difference is lower than 10%, the threshold is fixed, yielding an image where only the GFP area of the tumor cell mass is highlighted, creating a more accurate result. Different thresholds could be obtained for 0 hpi and 72 hpi, so the highest is selected to compare the evolution of the GFP area with time. The tolerance parameter establishes the percentage of decay with respect to area for a 0 threshold. (Fig. 2).

**Fig. 3:**
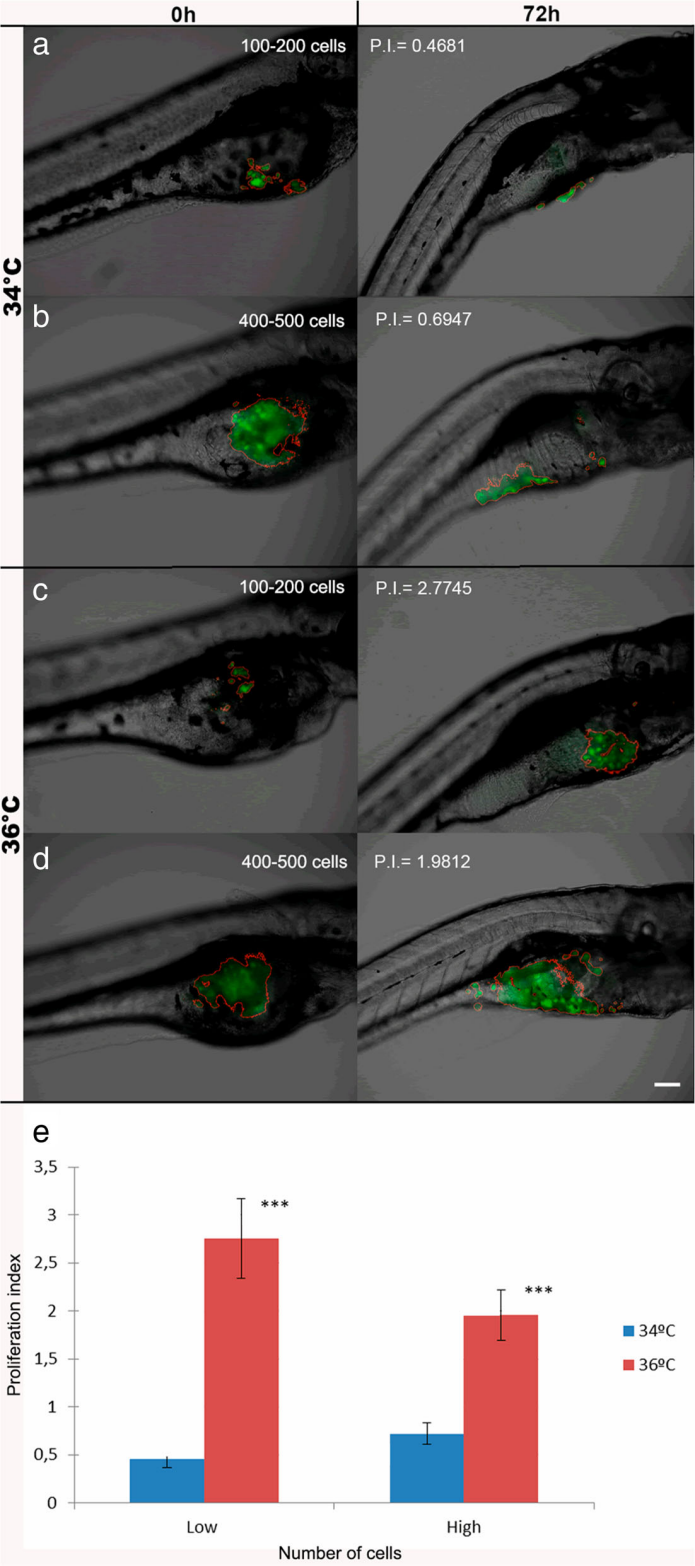
Proliferation assay in zebrafish injected with different cell numbers at 34 °C and 36 °C. **a**, **b** Initial injected cells proliferation index at an incubation temperature of 34 °C (**a**: 100-200 cells, P.I. = 0.4603; **b**: 400-500 cells; P.I. = 0.7196). **c**, **d** Initial injected cells proliferation index at an incubation temperature of 36 °C (**c**: 100-200 cells, P.I. = 2.7558; **d**: 400-500 cells, P.I. = 1.9558). Images are representative of each of the conditions assayed. All images are a superposition of a fluorescence field image over a bright field image. In all panels the left image is a 48 hpf or 0 hpi zebrafish embryo, and the right image is the same zebrafish embryo with 120 hpf or 72 hpi. Scale bar = 100 μm. P.I. = proliferation index. **e** Comparison between the initial number of cells injected (Low: 100-200 or High: 400-500) and their proliferation at two different temperatures tested (n_rep_ = 20-50, n_total_ = 207, ***p < 0.01)

While performing the experiments, we noticed that cell proliferation at the two temperatures tested varied depending on the initial cell load. To account for this variation, a ZFtool extension was developed to automatically count the number of cells prior to injection. For this, a microinjection with the same conditions of the experiment was performed over a microscope slide with low (100-200 cells) and high (400-500) cell numbers, photographed under a fluorescence microscope and analysed, after that, these cells were discarded [see Additional file 3: Figure S2]. Afterwards, the embryos were injected with the same conditions. When the comparison of initial injected cells is performed between the range of 100-200 and 400-500 cells/injection at 34 °C, the proliferation after 72 h remains the same, being not statistically significant when compared to 0 h. However, when the injections with 100-200 and 400-500 cells/injection are performed at 36 °C, the proliferation at 72 h is statistically significant, resulting in more proliferation of the injected cells when the number of those cells is in the range of 100-200 cells/injection (Fig. 3). This could be due to the space they have in the yolk and the sub estimation of the ZFtool when the number of cells is in the range of 400-500. In any case, the proliferation differences after 72 h, despite the initial number of injected cells, are statistically significant between 34 °C and 36 °C.

### In vivo comparative proliferation analysis at two different temperatures

**Fig. 4:**
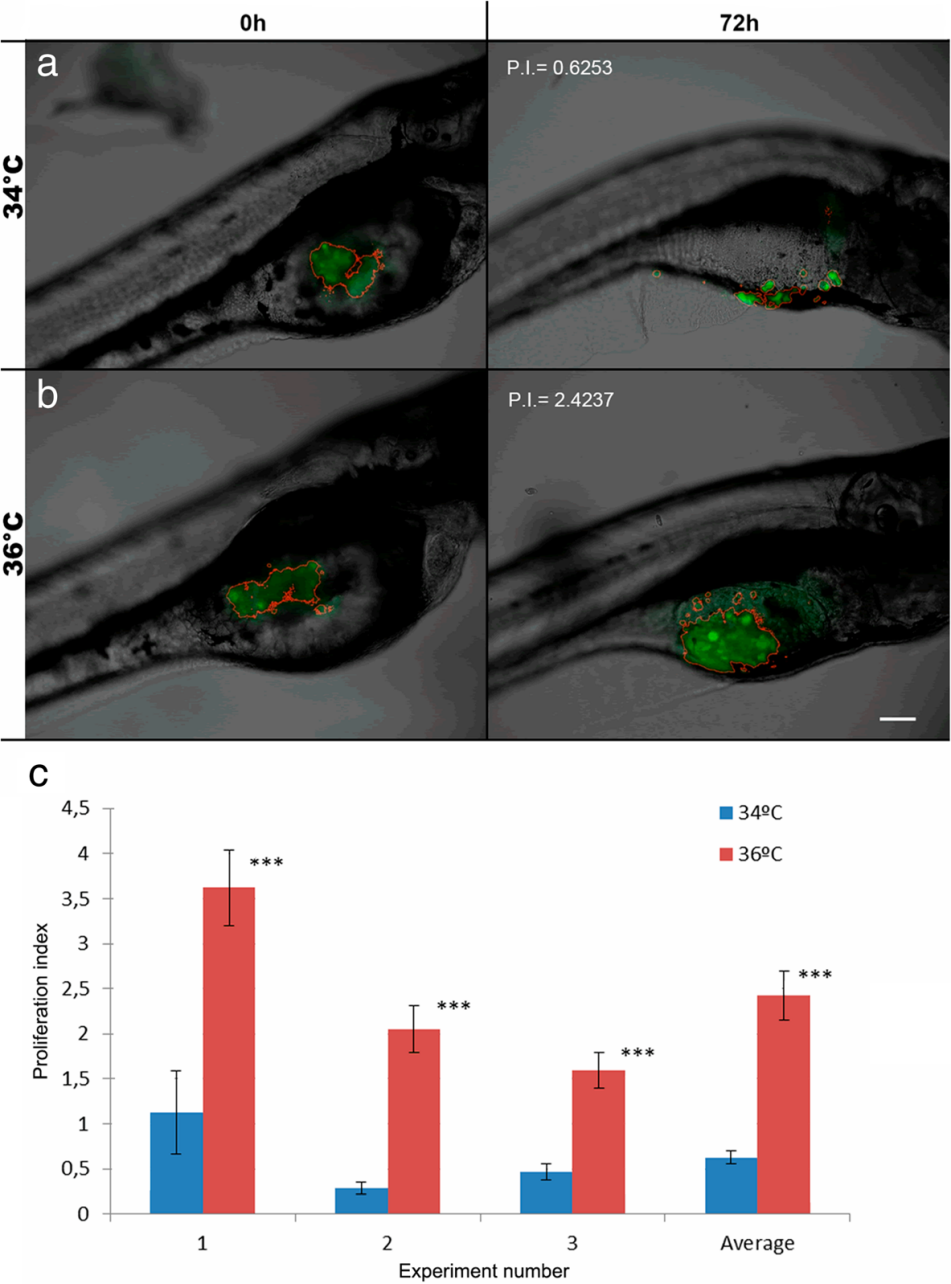
Cell proliferation inside the zebrafish embryos at the two conditions tested. **a** Zebrafish embryo incubation at 34 °C, analyzed with ZFtool, yielded a proliferation index of 0.6253 (**b**) Zebrafish embryo incubation at 36 °C analyzed with ZFtool yielded a proliferation index of 2.4237. Images are representative of each of the conditions assayed. All images are a superposition of a fluorescence field image over a bright field image. In all panels, the left image is a 48 hpf or 0 hpi zebrafish embryo, and the right image is the same zebrafish embryo with 120 hpf or 72 hpi. Scale bar = 100 μm. **c** Quantization of cell proliferation inside the embryos at the two temperatures tested in each experiment (34 °C-36 °C). (n_rep_ = 20-50, n_total_ = 207, ***p < 0.01)

The aim of this experiment was to test whether a better proliferation index exists at a temperature close to the human body (36.5 °C). Embryo post-transplant incubations were performed at two different temperatures (34 °C and 36 °C) to assay tumor cells behaving differently at both temperatures. Cultured HCT116 cells expressing GFP constitutively were microinjected into the embryos at 48 h post fecundation (hpf). After microinjection, embryos were photographed and placed in an incubator at 34 °C or 36 °C for 72 h. At 72 h post injection (hpi) embryos were photographed again. The results showed proliferation of HCT116 cells at 36 °C (2.4237). When compared to cells at 34 °C (0.6253), no proliferation was detected in this condition, but on the contrary, cell death appeared as a possibility, based on the lack of fluorescence (Fig. 4). These results are consistent compared with the results obtained in vitro.

### 5-fluorouracil toxicity test

A toxicity test was performed using the chemotherapeutic drug 5-Fluorouracil (5-FU) to establish a suitable concentration with the lowest toxicity possible at an effective therapeutic concentration for later use in our experiments. For this purpose, the OECD zebrafish toxicity protocol was performed using 0 h post fecundation (hpf) embryos and exposing them to different concentrations of 5-FU for 96 h at 26 °C [25]. Mortality was then determined in order to find the lowest toxicity of the compound over the embryos.

Recent studies indicated that the LC50 for the 5-FU following the OECD protocol at 26 °C for 120 hpf exposed embryos is 2222 mg/L or 17,082 μM [26].

The results of the OECD protocol at 26 °C showed that the concentrations tested were not sufficiently toxic enough to calculate the LC50. The aim of this toxicity test was not the calculation of the LC50 of the 5-FU, but to find the concentration at which the mortality of the fish was acceptable for our experiments and also effective against the injected cells (HCT116). The concentration with the greatest embryo survival (500 μM), could be determined at 26 °C [see Additional file 4: Figure S3].

Due to the lack of toxicity of 5-FU at 26 °C with the concentrations tested, the same experiment was performed with the final conditions of our experiments: 48 hpf embryos, instead of 0 h, for a period of 96 h at 36 °C. The same concentrations were tested, and the ideal concentration to assure the survival of the embryos was again at 500 μM. At 36 °C and 48 hpf, the mortality of the embryos using this compound was higher than at 26 °C and 0 hpf (Table 2).

**Table 2:** Toxicity test and mortality rates at 36 °C from 48 hpf to 144 hpf

Conc.^a^	5-FU	IC^b^	5-FU	IC^b^
Control -	0/24		0/24	
24 h	34 °C		36 °C	
250 μM	0/20	0/4	0/20	0/4
500 μM	0/20	0/4	0/20	0/4
1000 μM	0/20	0/4	0/20	0/4
1500 μM	0/20	0/4	1/20	0/4
2000 μM	1/20	0/4	2/20	0/4
48 h	34 °C		36 °C	
250 μM	0/20	0/4	2/20	0/4
500 μM	0/20	0/4	0/20	0/4
1000 μM	0/20	0/4	5/20	0/4
1500 μM	2/20	0/4	5/20	0/4
2000 μM	2/20	0/4	11/20	0/4
72 h	34 °C		36 °C	
250 μM	0/20	0/4	1/20	0/4
500 μM	0/20	0/4	0/20	0/4
1000 μM	0/20	0/4	3/20	0/4
1500 μM	1/20	0/4	3/20	0/4
2000 μM	1/20	0/4	10/20	0/4
96 h	34 °C		36 °C	
250 μM	0/20	1/4	2/20	0/4
500 μM	0/20	0/4	0/20	0/4
1000 μM	0/20	1/4	5/20	0/4
1500 μM	2/20	0/4	6/20	1/4
2000 μM	3/20	0/4	14/20	0/4

^a^*Conc* concentration, ^b^*IC* internal control. Negative control embryos were assayed in a separate 24 well plate. Additionally, four negative internal controls were placed in 4 of the 24 wells in each 5-FU treated plate being the other 20 wells 5-FU treatment

### ZFtool analysis of anti-tumor drug effectiveness

**Fig. 5:**
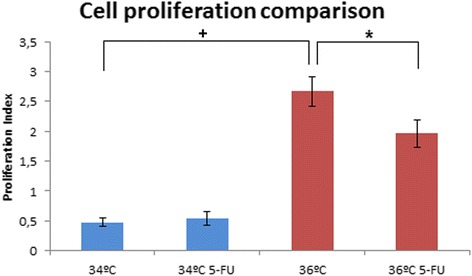
Cell proliferation inside the zebrafish embryos between 34 °C / 34 °C 5-FU and 36 °C / 36 °C 5-FU. Xenografted tumor cell proliferation at 34 °C / 34 °C 5-FU and 36 °C / 36 °C 5-FU. Proliferation at 34 °C = 0.4748 / 34 °C 5-FU = 0.5415 / 36 °C = 2.6652 / 36 °C 5-FU = 1.9592. Each column is an average representation of four independent experiments (n_rep_ = 81-102, n_total_ = 300, *p < 0.01; ^+^*p* < 0.01)

To determine the effect of the anti-tumor drug 5-FU on the injected cells at two different temperatures, an experiment was performed with the HCT116 colorectal cancer cell line. For this, 48 hpf HCT116 injected embryos were photographed and placed individually in 24-well plates with 2 mL SDTW/well and incubated at 34 °C and 36 °C for 24 h. After the incubation finished, embryos were photographed again to check the injected cells, and embryos without any were discarded. At this time, the embryos were transferred to 24-well plates containing: 2 mL SDTW/well and 500 μM 5-FU (DMSO final concentration 1%) or 2 mL SDTW/well with 1% DMSO (control fish). Final concentration of DMSO was used to dissolve the 5-FU, with no toxic effects as previously reported [27] and assayed in our laboratory to test the conditions in our embryos (data not shown). The fish were returned to the incubator at 34 °C or 36 °C for another 48 h. At 72 hpi, the embryos were photographed again and analysed with ZFtool [see Additional files 5 and 6: Figures S4 and S5]. The results obtained showed a reduction in the injected tumor cells at 36 °C compared to the controls, nevertheless no reduction was observed at 34 °C. At 34 °C the control group showed a proliferation index of 0.4748, while the 5-FU treated fish had a proliferation ratio of 0.5415, being the difference not statistically significant between control and treated embryos. At 36 °C the control group showed a proliferation ratio of 2.6653, while the 5-FU treated fish had a proliferation ratio of 1.9592. Again, the proliferation index performing this analysis was statistical significant. The statistical analysis demonstrated significant differences between the control and the treated group at this temperature (Fig. 5).

## Discussion

Model organisms as zebrafish have become a very important tool in the study of human diseases in recent years. Zebrafish, due to its characteristics and advantages, has emerged as an ideal model to study the behavior of different types of cancers and to test new chemotherapeutic compounds [28]. While the yolk does not provide the ideal microenvironment for tumor cells it is the suitable place to inject the cells to rapidly test chemotherapeutic compounds. Even this, enhancements of the xenotransplantation technique are required, together with accurate imaging analysis software to verify the fate of the cells inside the zebrafish embryo assuring a rapid analysis of xenotransplanted human cells when exposed to different treatments. This study describes an improvement in the xenotransplantation conditions in relation to temperature and the establishment of the injected cells combined with ZFtool image analysis.

Different authors reported normal development of zebrafish embryos up to 35.5 °C [29, 30], but a range of temperatures was tested in order to reduce the mortality of the embryos [see Additional file 1: Figure S6]. Some authors noted that more assays would be needed to check the proliferation, migration, and response of the cells to drugs at higher temperatures despite the potential increase in mortality [19].

We have set the temperature of the cells xenotransplanted into the zebrafish embryos closer to human temperature by raising the temperature from 28 °C (normal temperature at which zebrafish embryos develop) to 36 °C, with no significant change in mortality and sporadic developmental defects (curvature), due to the higher temperature, on the surviving embryos at 3 days post injection. Embryo incubation temperature is important to test the effects of anticancer drugs [9, 31], otherwise the temperature could affect the proliferation rate of the injected cells, and the effect of the drug is underestimated. The results in this study clearly show that the proliferation of injected cells and their response to anticancer drugs is better at 36 °C than at 34 °C; 36 °C being the temperature closer to their optimal growth temperature of 37 °C [8].

The number of injected cells is very relevant in terms of the proliferation and migration of these cells and should be considered for improved xenotransplantation and anticancer drug proliferation assays. The proliferation rate of the cells injected inside the embryos decays when the number of initial cells is insufficient at 34 °C. This may be due to cell-cell interactions: the cells injected appear to be isolated and cannot interact among themselves in order to proliferate properly. Nevertheless, even if the number of injected cells at 0 hpi is reduced, at 36 °C a higher proliferation rate of these cells exists compared to 34 °C, where the proliferation rate is absent. This previous point was assayed in vivo, demonstrating that despite the number of injected cells and mortality, 36 °C is an optimal temperature for cell growth. On the other hand, cell migration can also be modified, depending on the number of cells injected. Cells will not be able to migrate when the number of injected cells is insufficient. It is reported that 400 cells is the optimal number of injected cells to study these behaviors. Our cell line HCT116 remained in the yolk of the embryo from 0 hpi to 72 hpi, consistent with other authors that used the HCT116 cell line. This cell line has a low dissemination ratio [9, 22].

In in vitro studies, other authors have performed in vitro proliferation assays with the 3-(4,5-dimethylthiazol-2-yl)-2,5-diphenyl tetrazolium bromide colorimetric assay (MTT). The initial cell density seeded on the plates was the same for each experiment, so there was no assessment of how the proliferation could change with different concentrations of the initial cells seeded [9, 20]. In this study, we show that at least for the cell line HCT116, the temperature and number of initialy seeded cells are critical factors for the proliferation of injected cells.

Together with the work done for the improvement of zebrafish xenotransplantation, a method inside ZFTool was developed to measure the cell proliferation inside the yolk of the embryo. This method was designed to fill the gap present in the current methodology that does not adequately quantify cell proliferation at different times in vivo. For example, flow cytometry is not sensitive enough to quantify the number of cells in dispersed embryos [32], and software used by other authors, such as ImageJ or Photoshop, does not automatically quantify proliferation in order to compare high number of fishes per experiment, since they require a higher amount of user intervention per fish.

In summary, we demonstrated that at 36 °C, a better proliferation rate exists for the injected cells inside the embryos, with no significant mortality changes compared with 34 °C. Our results also reveal a correlation between the number of initially injected cells and the proliferation ratio when comparing the two different temperatures. In addition, we used a new image analysis software, the ZFtool, which improves tumor cell quantification in vivo with accuracy and speed. One of the future challenges will be the quantification of these cells with a 3D method with much greater accuracy, reaching the count of each cell individually.

## Conclusions

This study demonstrates that human colorectal cancer cell line HCT116 injected into zebrafish embryos has a better proliferation index at 36 °C rather than at 34 °C. Furthermore, 36 °C is the most suitable temperature for testing chemotherapeutic drugs like the 5-Fluorouracil.

## Additional files

Additional file 1: Figure S6. Revision of parameters regarding xenotransplantation conditions. (PDF 296 kb)

Additional file 2: Figure S1. ZFtool automatically elimination of fish autofluorescence. ZFtool software detects all the green pixels in the image (red/pink line) but eliminates all those pixels corresponding to fish autofluorescence and keeps pixels above an established threshold (blue line). (TIFF 7994 kb)

Additional file 3: Figure S2. Automated counting of cells. This image shows the process of the software to count the cells on the microscope slide performed before the injection of the zebrafish embryos. (A) Fluorescent image of low cell number. (B) Cells of the A image counted (179). (C) Fluorescent image of high cell number. D: Cells of the C counted (404). Scale bar = 100 μm. (TIFF 1115 kb)

Additional file 4: Figure S3. OECD protocol toxicity results. (DOCX 18 kb)

Additional file 5: Figure S4. Cell proliferation inside the zebrafish embryos at 34 °C and 34 °C with 5-FU (A) Zebrafish embryo incubation at 34 °C analyzed with ZFtool yielding a proliferation index of 0.4748. (B) Zebrafish embryo incubation at 34 °C, with 5-FU analyzed with the ZFtool yielding a proliferation index of 0.5415. All images are a superposition of a fluorescence field image over a bright field image. In all panels, the left image is a 48 hpf or 0 hpi zebrafish embryo, and the right image is the same zebrafish embryo with 120 hpf or 72 hpi. Scale bar = 100 μm. (TIFF 8180 kb)

Additional file 6: Figure S5. Cell proliferation inside the zebrafish embryos at 36 °C and 36 °C with 5-FU (A) Zebrafish embryo incubation at 36 °C analyzed with ZFtool yielding a proliferation index of 2.6653. (B) Zebrafish embryo incubation at 36 °C, with 5-FU analyzed with the ZFtool yielding a proliferation index of 1.9592. All images are a superposition of a fluorescence field image over a bright field image. In all panels, the left image is a 48 hpf or 0 hpi zebrafish embryo, and the right image is the same zebrafish embryo with 120 hpf or 72 hpi. Scale bar = 100 μm. (TIFF 10158 kb)

## Abbreviations

5-FU: 5-Fluorouracil
dpf: Days post-fertilization
GFP: Green fluorescence protein
GMV: GFP medium valor
hpf: Hours post-fecundation
hpi: Hours post-injection
nGFP: Number of GFP pixels
