# Supplementary material for: Improving zebrafish embryo xenotransplantation conditions by increasing incubation temperature and establishing a proliferation index with ZFtool
Source: BMC Cancer. 2018 Jan 2;18:3. doi: 10.1186/s12885-017-3919-8 (PMC5748948; doi:10.1186/s12885-017-3919-8)
Supplement: Supplementary file 1 — Revision of parameters regarding xenotransplantation conditions. (PDF 296 kb) [file 12885_2017_3919_MOESM1_ESM.pdf]

Paper	Temperature	Nº cells	Image Analysis	Xenograft time
**Lee *et al.* 2005**	31ºC	1-100	Adobe Photoshop	Blastula-5dpf
**Haldi *et al.* 2006**	35ºC (1h at 28ºC)	50-200	Adobe Photoshop	2dpf-7dpf
**Nicoli *et al.* 2007**	n/a	1000-2000	Image Pro Plus	2dpf-3dpf
**Nicoli & Presta 2007**	28ºC (24-48h)	4-10 nanoliter	Image Pro Plus	2dpf
**White *et al.* 2008**	28ºC adultos	100.000 Kidney Marrow cells (and 200.000 blood cells), 200.000 Melanoma	Image J. Software, NIH, Bethesda, USA	4 weeks
**Harfouche *et al.* 2009**	n/a	500	NIS-Element software (Nikon Instruments)	n/a
**Lee *et al.* 2009**	28ºC	100-500	n/a	2dpf-8dpf
**Marques *et al.* 2009**	35ºC (1h at 31ºC)	n/a	n/a	2dpf-5dpf or 7 dpi
**Stoletov *et al.* 2010**	35,5ºC	30-100	Confocal	Hours-1 día
**Wagner *et al.* 2010**	28ºc 1h and 35ºC after	n/a	n/a	Blastula - 5dpf
**Corkery *et al.* 2011**	35ºC (1h at 28ºC)	25-50	Image J, NIH, Bethesda, MD, USA	2dpf-9dpf (7dpi)
**Eguiara *et al.* 2011**	34ºC (2h at 28ºC)	500	n/a	2dpf-9dpf (7dpi)
**Moshal *et al.* 2011**	31ºC 1h and 28,5ºC for treatment	600-800	NIS-Element AR software (Nikon Instruments)	1dpf-3dpf (2dpi)
**Pruvot *et al.* 2011**	34ºC (1h at 28ºC)	50-200	Adobe Photoshop	2dpf-6dpf (4dpi)
**Zhao *et al.* 2011**	28ºC	50-100	Axiovision rel. 4.8 software	2dpf-8dpf (6dpi)
**Benyumov *et al.* 2012**	28.5ºC	100	AxioVision software (Release 4.7.2)	Blastula - 2dpi
**Ghotra *et al.* 2012**	34ºC	100	Image-Pro Plus-based software from Media Cybernetics	2dpf-6dpi

**He *et al.* 2012**	34ºC	50-400	Zeiss ZEN2009 software or ImageJ software, Adobe CS4.	2dpf-6dpi (4days)
**Jung *et al.* 2012**	28, 31 and 35 (31ºC)	25, 100, 200 (better dissemination with 200)	Cell count before injection, disgregation and invert microscopy for counting	2dpf-6dpf (4dpi)
**Drabsch *et al.* 2013**	33ºC	400	Adobe Photoshop CS4.	2dpf-8dpf (6dpi)
**Jo DH *et al.* 2013**	n/a	20 y 100	ImageJ Software	2dpf-6dpf (4dpi)
**Spaink *et al.* 2013**	34ºC	400 minimum (500)	Linutop, COPAS XL cell count + epifluorescence, Perl package software.	256 cells - 5dpf / 2-4hpf - 8dpf (6dpi)
**Teppo *et al.* 2013**	n/a	n/a	ZebIAT	2dpf-5dpf (3dpi)
**Yang *et al.* 2013**	35ºC	200-1000 (300)	Image J. Software, NIH, Bethesda, USA. Adobe Photoshop.	2dpf-X
**Teng *et al.* 2013**	34ºC	300	ImageJ/Fiji.	2dpf-4/6dpf
**Ban *et al.* 2014**	34ºC	500	Image J	2dpf-6dpf (4dpi)
**Bansal *et al.* 2014**	33ºC	10-500	n/a	2dpf-14dpf (12dpi)
**Bentley *et al.* 2014**	35ºC	15-20	n/a	2dpf-6dpf/9dpf (4dpi/7dpi)
**Ent *et al.* 2014**	34ºC	24-44	Image J / Image analysis (ImagePro Analyzer 7.0)	2dpf-8dpf (6dpi)
**Orlova *et al.* 2014**	33ºC	100/group	Adobe Photoshop CS4	Early blastula (256/512 cell) / 2dpf-X
**Zhang *et al.* 2014 (1)**	32ºC	100-200	MetaXpress Software	2dpf-9dpf (7dpi)

**Zhao *et al.* 2014**	28ºC	n/a	n/a	2dpf-6dpf (4dpi)
