# Supplementary figures and images for: Improving zebrafish embryo xenotransplantation conditions by increasing incubation temperature and establishing a proliferation index with ZFtool
Source: BMC Cancer. 2018 Jan 2;18:3. doi: 10.1186/s12885-017-3919-8 (PMC5748948; doi:10.1186/s12885-017-3919-8)

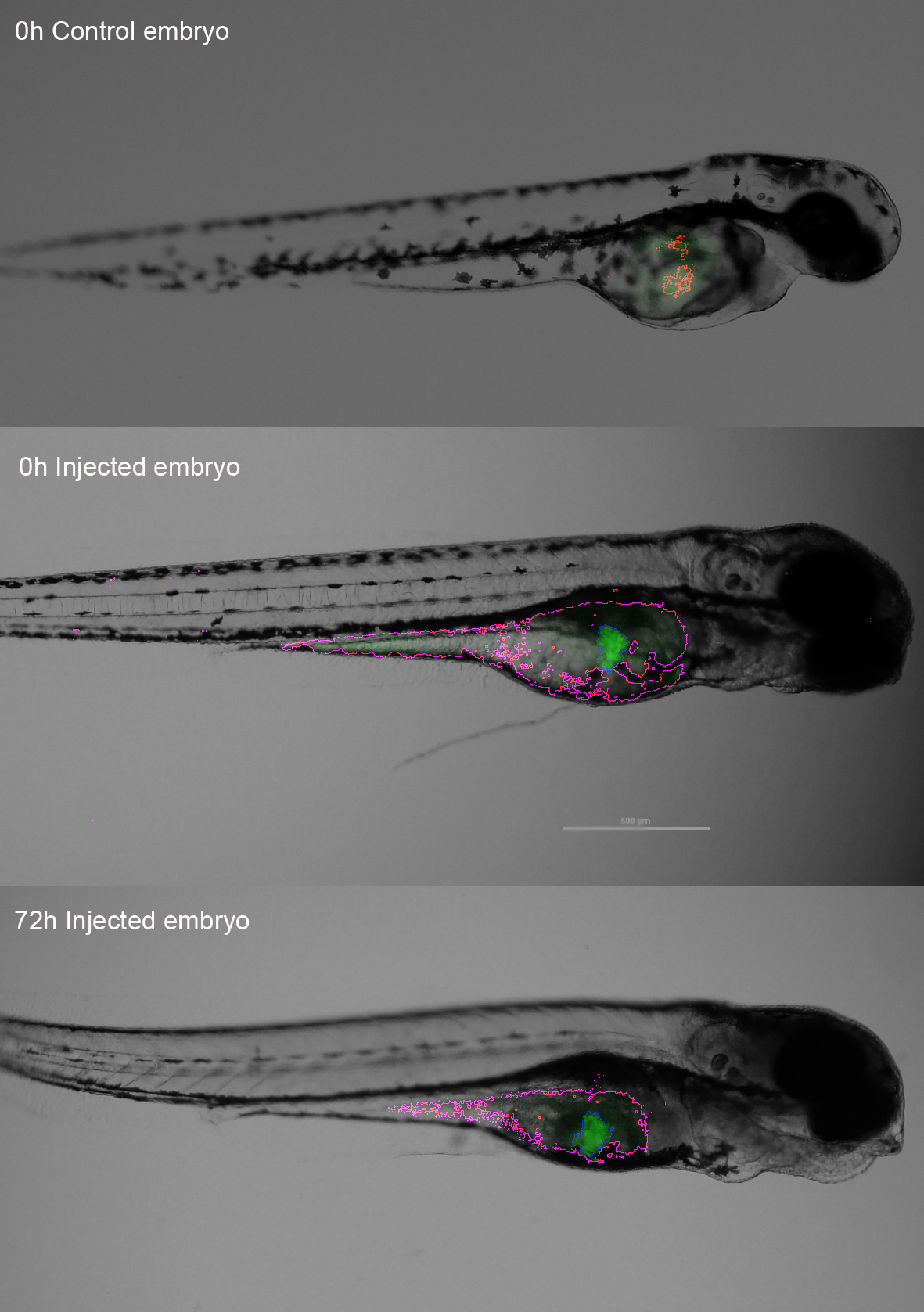

Supplement: Supplementary file 2 — ZFtool automatically elimination of fish autofluorescence. ZFtool software detects all the green pixels in the image (red/pink line) but eliminates all those pixels corresponding to fish autofluorescence and keeps pixels above an established threshold (blue line). (TIFF 7994 kb) [file 12885_2017_3919_MOESM2_ESM.tif]

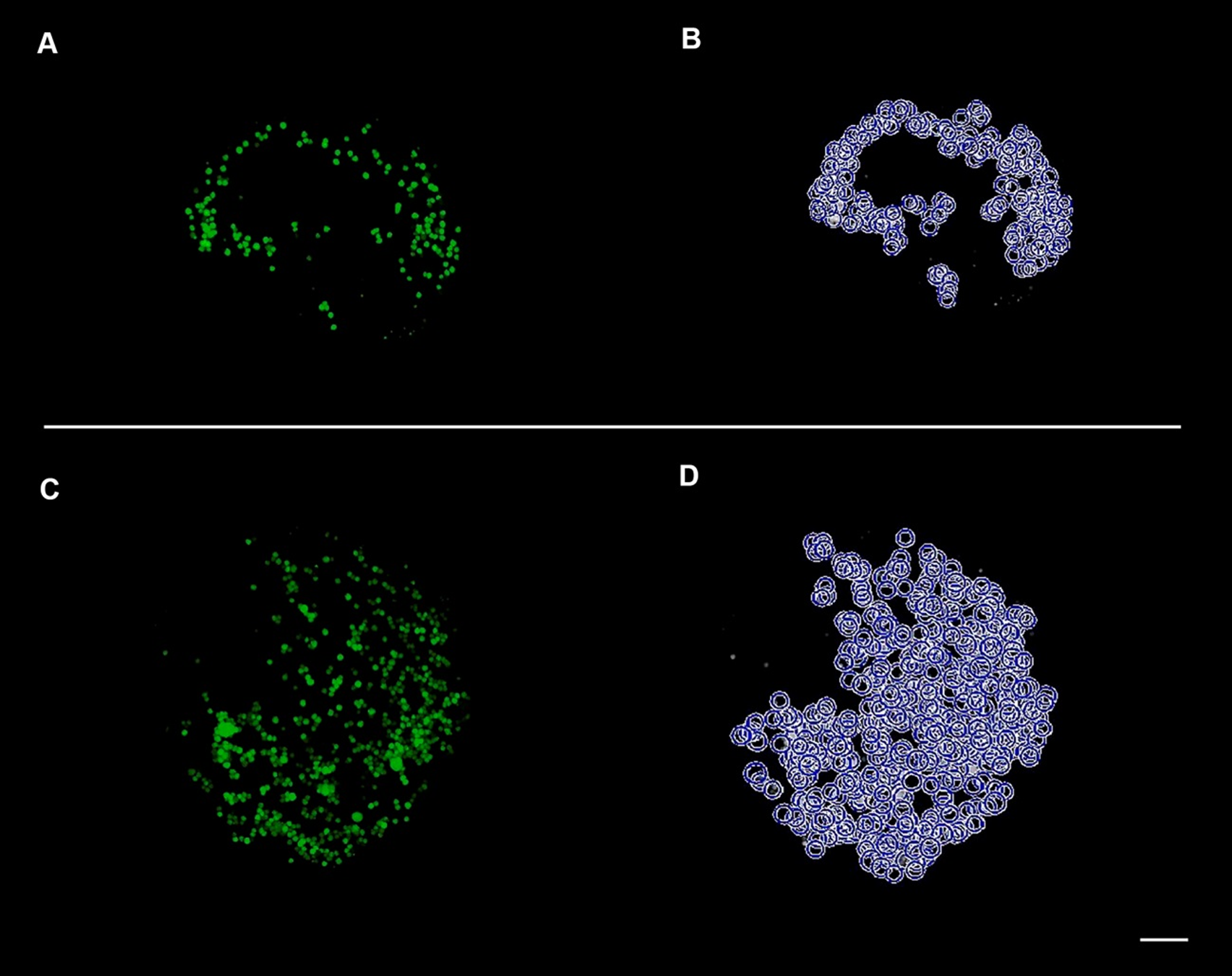

Supplement: Supplementary file 3 — Automated counting of cells. This image shows the process of the software to count the cells on the microscope slide performed before the injection of the zebrafish embryos. (A) Fluorescent image of low cell number. (B) Cells of the A image counted (179). (C) Fluorescent image of high cell number. D: Cells of the C counted (404). Scale bar = 100 μm. (TIFF 1115 kb) [file 12885_2017_3919_MOESM3_ESM.tif]

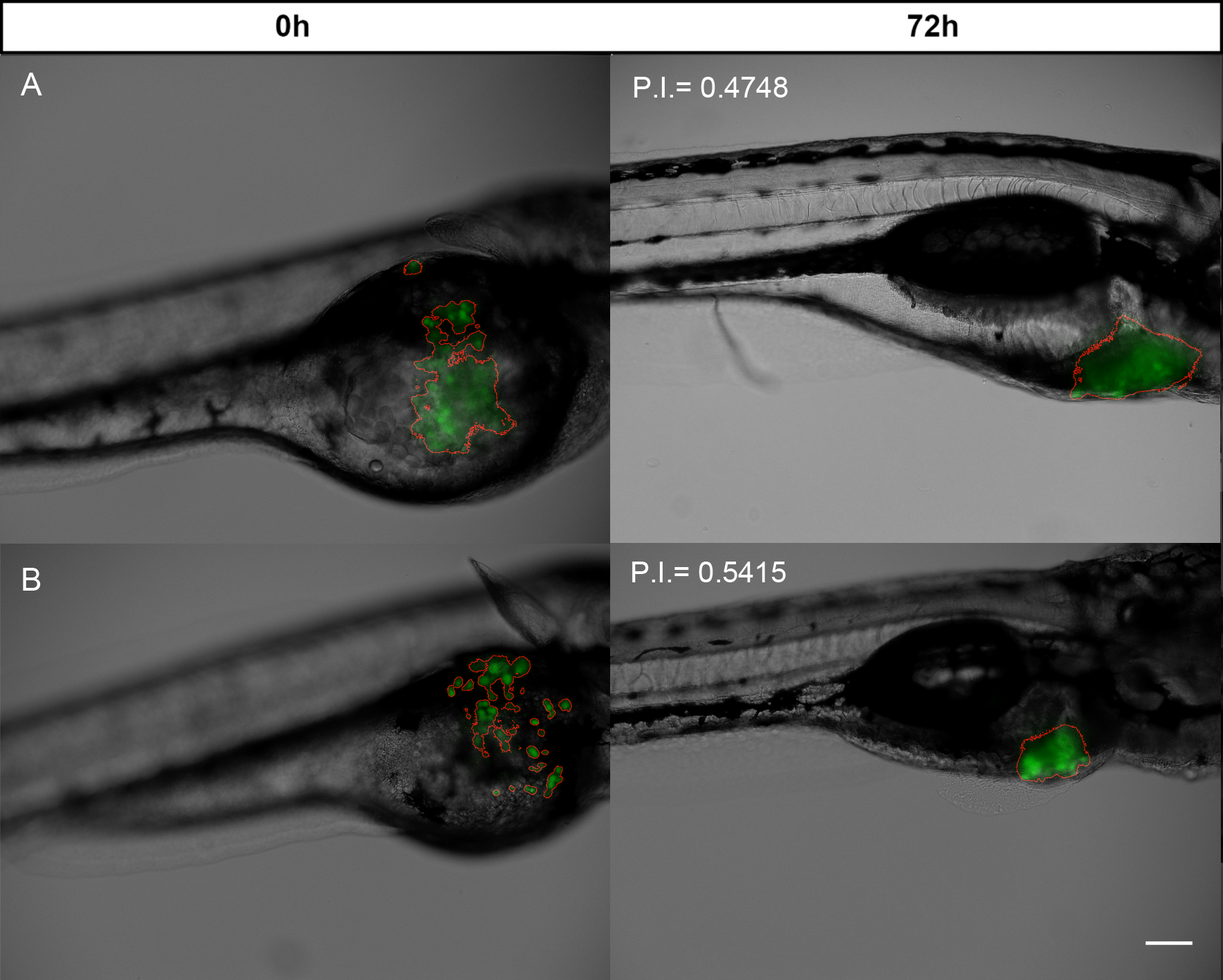

Supplement: Supplementary file 5 — Cell proliferation inside the zebrafish embryos at 34 °C and 34 °C with 5-FU (A) Zebrafish embryo incubation at 34 °C analyzed with ZFtool yielding a proliferation index of 0.4748. (B) Zebrafish embryo incubation at 34 °C, with 5-FU analyzed with the ZFtool yielding a proliferation index of 0.5415. All images are a superposition of a fluorescence field image over a bright field image. In all panels, the left image is a 48 hpf or 0 hpi zebrafish embryo, and the right image is the same zebrafish embryo with 120 hpf or 72 hpi. Scale bar = 100 μm. (TIFF 8180 kb) [file 12885_2017_3919_MOESM5_ESM.tif]

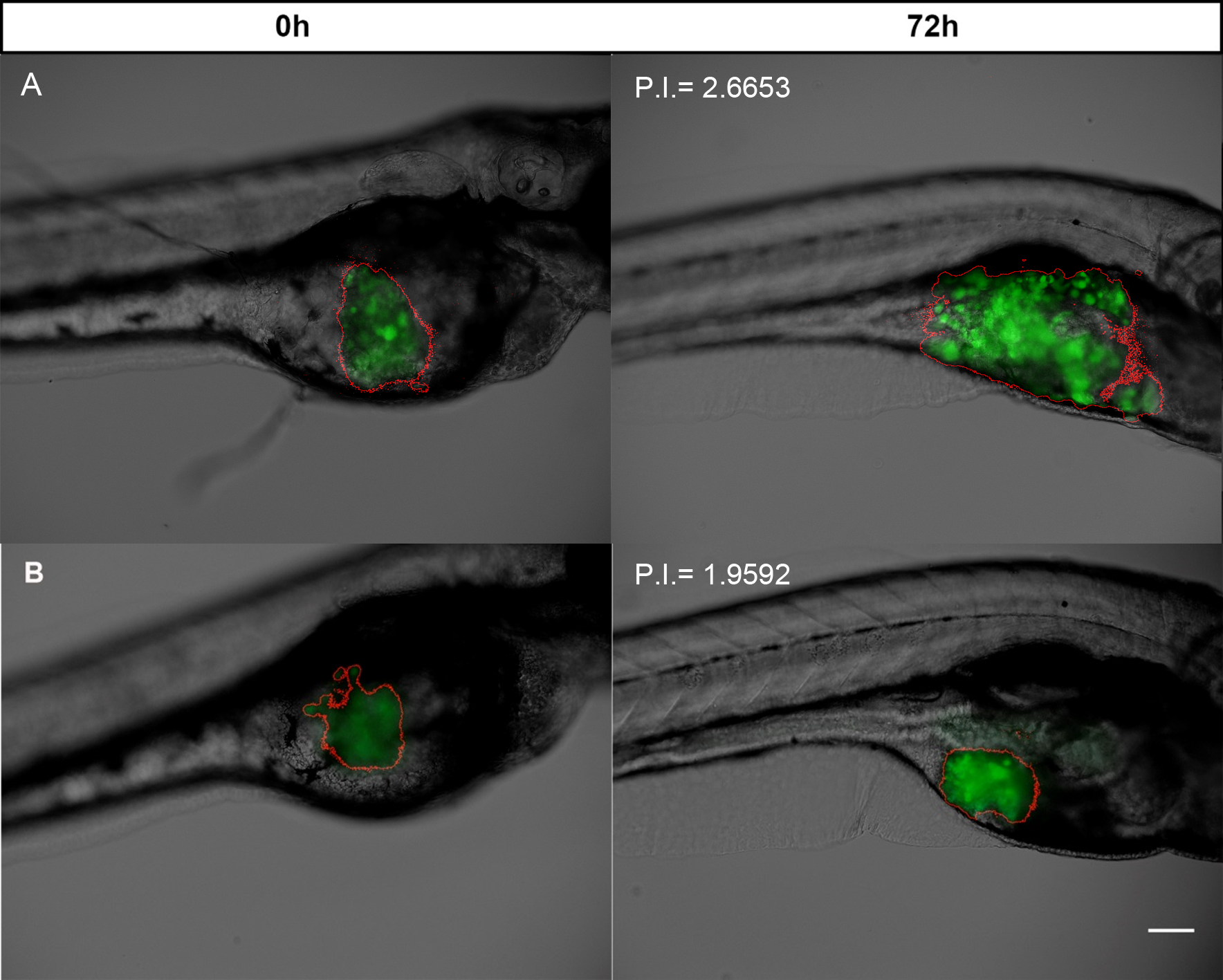

Supplement: Supplementary file 6 — Cell proliferation inside the zebrafish embryos at 36 °C and 36 °C with 5-FU (A) Zebrafish embryo incubation at 36 °C analyzed with ZFtool yielding a proliferation index of 2.6653. (B) Zebrafish embryo incubation at 36 °C, with 5-FU analyzed with the ZFtool yielding a proliferation index of 1.9592. All images are a superposition of a fluorescence field image over a bright field image. In all panels, the left image is a 48 hpf or 0 hpi zebrafish embryo, and the right image is the same zebrafish embryo with 120 hpf or 72 hpi. Scale bar = 100 μm. (TIFF 10158 kb) [file 12885_2017_3919_MOESM6_ESM.tif]
