# Supplementary material for: Improving zebrafish embryo xenotransplantation conditions by increasing incubation temperature and establishing a proliferation index with ZFtool
Source: BMC Cancer. 2018 Jan 2;18:3. doi: 10.1186/s12885-017-3919-8 (PMC5748948; doi:10.1186/s12885-017-3919-8)
Supplement: Supplementary file 4 — OECD protocol toxicity results. (DOCX 18 kb) [file 12885_2017_3919_MOESM4_ESM.docx]

**Table 2. OECD protocol toxicity results (0 h-96 h).**

| **24 h** | **R1** | | **R2** | | **R3** | |
| --- | --- | --- | --- | --- | --- | --- |
| Control - | 2 | | 2 | | 0 | |
| **Conc.** | **5-FU** | **IC** | **5-FU** | **IC** | **5-FU** | **IC** |
| 250 µM | 0 | 1 | 0 | 0 | 3 | 0 |
| 500 µM | 0 | 0 | 1 | 0 | 0 | 1 |
| 1000 µM | 0 | 0 | 1 | 2 | 1 | 0 |
| 1500 µM | 0 | 1 | 3 | 0 | 2 | 0 |
| 2000 µM | 1 | 1 | 2 | 0 | 2 | 1 |

| **48 h** | **R1** | | **R2** | | **R3** | |
| --- | --- | --- | --- | --- | --- | --- |
| Control - | 6 | | 2 | | 2 | |
| **Conc.** | **5-FU** | **IC** | **5-FU** | **IC** | **5-FU** | **IC** |
| 250 µM | 0 | 1 | 0 | 1 | 3 | 0 |
| 500 µM | 0 | 0 | 1 | 0 | 0 | 2 |
| 1000 µM | 0 | 0 | 2 | 2 | 1 | 1 |
| 1500 µM | 0 | 2 | 3 | 0 | 2 | 0 |
| 2000 µM | 1 | 1 | 2 | 0 | 2 | 1 |

| **72 h** | **R1** | | **R2** | | **R3** | |
| --- | --- | --- | --- | --- | --- | --- |
| Control - | 6 | | 3 | | 2 | |
| **Conc.** | **5-FU** | **IC** | **5-FU** | **IC** | **5-FU** | **IC** |
| 250 µM | 0 | 1 | 0 | 1 | 3 | 2 |
| 500 µM | 0 | 0 | 1 | 0 | 0 | 2 |
| 1000 µM | 0 | 0 | 2 | 2 | 1 | 1 |
| 1500 µM | 0 | 2 | 3 | 0 | 2 | 0 |
| 2000 µM | 1 | 1 | 2 | 0 | 2 | 1 |

| **96 h** | **R1** | | **R2** | | **R3** | |
| --- | --- | --- | --- | --- | --- | --- |
| Control - | 6 | | 3 | | 2 | |
| **Conc.** | **5-FU** | **IC** | **5-FU** | **IC** | **5-FU** | **IC** |
| 250 µM | 0 | 1 | 0 | 1 | 3 | 2 |
| 500 µM | 0 | 0 | 1 | 0 | 0 | 2 |
| 1000 µM | 1 | 0 | 2 | 2 | 1 | 1 |
| 1500 µM | 0 | 2 | 3 | 0 | 2 | 0 |
| 2000 µM | 1 | 1 | 3 | 0 | 2 | 1 |

*****The number of dead embryos is shown in the table for each concentration of the compound. R1, R2, R3, Replica 1, 2, 3; Conc, concentration; IC, internal control.
